# Blocking the human common beta subunit of the GM-CSF, IL-5 and IL-3 receptors markedly reduces hyperinflammation in ARDS models

**DOI:** 10.1038/s41419-022-04589-z

**Published:** 2022-02-10

**Authors:** Hao Wang, Damon J. Tumes, Timothy R. Hercus, K. H. Yip, Christian Aloe, Ross Vlahos, Angel F. Lopez, Nick Wilson, Catherine M. Owczarek, Steven Bozinovski

**Affiliations:** 1 School of Health & Biomedical Sciences, RMIT University, Bundoora, VIC Australia; 2 Centre for Cancer Biology, SA Pathology and UniSA, Adelaide, Australia; 3 CSL Limited, Parkville, VIC Australia

**Keywords:** Acute inflammation, Innate immunity

## Abstract

Acute respiratory distress syndrome (ARDS) is triggered by various aetiological factors such as trauma, sepsis and respiratory viruses including SARS-CoV-2 and influenza A virus. Immune profiling of severe COVID-19 patients has identified a complex pattern of cytokines including granulocyte macrophage-colony stimulating factor (GM-CSF) and interleukin (IL)-5, which are significant mediators of viral-induced hyperinflammation. This strong response has prompted the development of therapies that block GM-CSF and other cytokines individually to limit inflammation related pathology. The common cytokine binding site of the human common beta (β_c_) receptor signals for three inflammatory cytokines: GM-CSF, IL-5 and IL-3. In this study, β_c_ was targeted with the monoclonal antibody (mAb) CSL311 in engineered mice devoid of mouse β_c_ and β_IL-3_ and expressing human β_c_ (hβ_c_Tg mice). Direct pulmonary administration of lipopolysaccharide (LPS) caused ARDS-like lung injury, and CSL311 markedly reduced lung inflammation and oedema, resulting in improved oxygen saturation levels in hβ_c_Tg mice. In a separate model, influenza (HKx31) lung infection caused viral pneumonia associated with a large influx of myeloid cells into the lungs of hβ_c_Tg mice. The therapeutic application of CSL311 potently decreased accumulation of monocytes/macrophages, neutrophils, and eosinophils without altering lung viral loads. Furthermore, CSL311 treatment did not limit the viral-induced expansion of NK and NKT cells, or the tissue expression of type I/II/III interferons needed for efficient viral clearance. Simultaneously blocking GM-CSF, IL-5 and IL-3 signalling with CSL311 may represent an improved and clinically applicable strategy to reducing hyperinflammation in the ARDS setting.

## Introduction

Acute respiratory distress syndrome (ARDS) is a hyperinflammatory disorder associated with an influx of activated myeloid cells into the pulmonary parenchyma and arteries that can contribute to fatal immunopathology. T cells at the inflammatory site produce several cytokines including granulocyte macrophage-colony stimulating factor (GM-CSF), which is strikingly elevated in fatal cases of COVID-19 [1] and capable of triggering a hyperinflammatory storm [2, 3]. GM-CSF can also regulate the intensity of neutrophilic lung inflammation [4, 5] and an activated neutrophil signature has been identified whereby neutrophil markers precede critical illness and predict mortality in COVID-19 [6]. Activated neutrophils release neutrophil extracellular traps (NETs), which are extruded chromatin fibres decorated with granule proteins including myeloperoxidase (MPO) and neutrophil elastase (NE) that normally entrap and kill pathogens. Excessive NET infiltrate in the lung and vasculature is thought to contribute to pathophysiology and mucosal cell death in severe COVID-19 patients [7–9].

The β common family of cytokines GM-CSF, interleukin (IL)-5 and IL-3 regulate myeloid cells by binding to heterodimeric receptors comprising a cytokine specific α-chain and a common β receptor (βc) that is the major signalling subunit. Mavrilimumab and Lenzilumab are monoclonal antibodies targeting the α-chain of GM-CSF receptor (GM-CSFR) and GM-CSF respectively. Both have a favourable safety profile and are showing promising therapeutic benefit in small COVD-19 clinical trials [10, 11], which support the advancement to larger clinical studies to clarify the significance of GM-CSF-dependent immunopathogenesis. An alternative strategy to blocking GM-CSF is to target the shared βc subunit, with the potential advantage of also blocking two other pro-inflammatory cytokines, IL-3 and IL-5 [12]. The role of type-2 immunity and IL-3 requires further investigation in severe COVID-19 patients, however there may be a pathogenic role for type-2 immunity at later stages of infection [13]. Intriguingly, patients who developed severe disease displayed a complex maladapted immune profile accompanied by an increase in cytokines linked to cytokine release syndrome (e.g. IL-6), type-1 cytokines (e.g. IL-12) and type-2 cytokines (e.g. IL-5) [13]. IL-5 is a central mediator of eosinophil maturation and survival and eosinophilic pneumonia can occur consequent to viral infection [14–16].

To date, no study has tested the efficacy of an antagonist that targets the common cytokine binding site within the human β_c_ receptor in an ARDS or viral pneumonia model, as species specificity has precluded this. To overcome this issue, a humanised transgenic mouse model expressing the human β_c_ receptor has recently been developed [17] to test the efficacy of a human monoclonal antibody (CSL311) in the pre-clinical disease setting. CSL311 blocked human βc receptor signalling with high potency [12, 18–21] and also strongly inhibited the development of experimental allergic contact dermatitis [17]. In this study, we specifically tested whether CSL311 can reduce immunopathology and hyperinflammation in two separate pre-clinical models of ARDS/ALI and viral pneumonia. We demonstrate that β_c_ receptor signalling drives lung inflammation and injury in a manner that is potently inhibited by CSL311 treatment.

## Methods

### Animals

All animal experiments were approved at RMIT University (animal ethics approval #1902) in accordance with the NHMRC of Australia and ARRIVE guidelines. Humanised transgenic mice were used, where the endogenous mouse βc and β_IL-3_ receptor have been knocked out and replaced with the human (h)βc receptor [17]. Immune cells isolated from hβ_c_Tg mice respond to mouse GM-CSF and IL-5 but not to IL-3 under in vitro conditions [17]. This supports that the mouse GM-CSF and IL-5 α receptor subunits efficiently interact with the human β_c_ receptor subunit, whereas the mouse IL-3Rα subunit does not interact efficiently with the human β_c_ receptor subunit [17]. Eight-to-twelve-week-old male and female hβcTg mice bred at RMIT University were used for experiments. To induce ALI, hβc mice were instilled intranasally with 1–10 µg LPS (*Escherichia coli* serotype O26:B6, Sigma, US) under light isoflurane anaesthesia and saline was used as vehicle control. CSL311, a human monoclonal antibody that blocks β_c_ cytokine binding and signalling [17, 18] and matching isotype control antibody [17] were used to in this study. CSL311 (3–50 mg/kg) or isotype control mAb was administered via intravenous injection 3 h prior to LPS challenge. Pulse oximetry was used to assess peripheral blood oxygenation over a 72-hour period using a MouseSTAT® Jr. Pulse Oximeter (Kent Scientific, US). Mice were culled 24 h and 72 h respectively after LPS treatment by pentobarbital overdosing for post-mortem analysis. To evaluate CSL311 in the viral pneumonia setting, hβcTg mice were intranasally infected with influenza A virus (IAV, HKx31, H3N2 strain, 10^4^ PFU) under light isoflurane anaesthesia. Four days after IAV infection, mice were treated with CSL311 (50 mg/kg) or isotype control mAb via intravenous injection and culled on day 6 post infection.

### BAL, blood and biochemical assays

Blood was collected by cardiac puncture for haematologic analysis (Cell-Dyn Emerald, Abbott Laboratories, US). Bronchoalveolar lavage (BAL) was performed as previously described [4, 22] and BAL cells were enumerated. Briefly, cytospins were prepared and stained with Shandon™ Kwik-Diff™ Stains (Thermofisher Scientific, US) for differential cell counts. Lungs were perfused with ice-cold PBS to remove residual blood. The superior lobe of the lungs was removed for flow cytometry analysis and the left lobe was excised and fixed in 10% neutral-buffered formalin for histology. The rest of the lungs were snap-frozen in liquid nitrogen prior to −80 °C storage. Myeloperoxidase (MPO) activity in lung tissue homogenates was performed as previously described [23]. Total protein levels in the BAL fluid were quantified using the BCA protein assay (Thermofisher Scientific, US) and dsDNA levels in the BAL fluid were measured using the Quant-iT™ PicoGreen™ dsDNA Assay Kit (Thermofisher Scientific, US).

### Histology and immunohistochemistry

The left lobes of the lungs were fixed in 10% neutral-buffered formalin for 24 h before storing in 70% ethanol. Tissues were processed, paraffin-embedded and sectioned vertically at a thickness of 4 µm. Coronal sections that uniformly contain bronchi and >8 airways were then stained with hematoxylin and eosin (H&E) and scanned using a VS120 Slidescanner (Olympus, Japan). Lung injury was assessed in a blinded manner on a scale of 0–3 (none to severe) in peribronchial, perivascular and interstitial/alveolar regions individually based on the degree of inflammatory cell infiltration, epithelial/endothelial destruction, and alveolar septal thickening as previously described [24] where total scores were presented. NET staining on lung sections was performed as previously described [25] using primary goat anti-mouse MPO antibody (1:40 dilution, AF3667, R&D Systems, US) and rabbit anti-mouse citrullinated histone antibody (1:100, ab5103 Abcam, UK). Sections were then labelled with Alexa Fluor 568 donkey anti-rabbit IgG (1:200) and Alexa Fluor 488 donkey anti-goat IgG (1:200) secondary antibodies and counterstained with DAPI (1:1000) (all from Thermofisher Scientific, US). Whole slides were scanned using a VS120 Slidescanner (Olympus, Japan). NETs were recognised by the yellow staining after fluorescence channel overlay.

### Flow cytometry

Following BAL, the superior lobes of the lungs were finely minced and digested in Liberase TM (Sigma, US) at 37 °C with constant shaking. Single cell suspension was prepared by passing the digested tissue through a 25 G needle and then a 40 µm cell strainer. Lung cells were pelleted, and red blood cells were lysed with ACK lysis buffer. After blocking with CD16/CD32 antibody, cells were then stained with a myeloid cell antibody cocktail containing FITC-CD45, PE-Siglec F, APC-F4/80, eFluor 450-CD11b, PE/Cy7-CD11c, PerCp/eFluor710-Ly6G and LIVE/DEAD™ Fixable Yellow Dead Cell Stain, or a lymphoid cell antibody cocktail containing APC/eFluor 780-CD45, PerCP/Cyanine5.5-CD3e, PE/Cy7-NK-1.1 (BD, US), PE-CD8a (BioLegend, US), PE/eFluor 610-CD4, Alexa Fluor 488-FOXP3 (BioLegend, US) and LIVE/DEAD™ Fixable Violet Dead Cell Stain as previously described [23, 26, 27]. A Foxp3 / Transcription Factor Staining Buffer Set was used for cell permeabilization before Foxp3 staining. After staining, cells were fixed with an IC Fixation Buffer before analysed on a BD FACSAria. All antibodies and reagents were from Thermofisher Scientific, US unless otherwise stated.

### Quantitative reverse transcription PCR (RT-qPCR)

Total RNA was extracted from the snap-frozen lung lobes using a RNeasy kit (Qiagen, Germany), from which cDNA was prepared with a High Capacity cDNA Kit (Thermofisher Scientific, US). qPCR was performed using bioinformatically validated TaqMan probes (Thermofisher Scientific, US). The threshold cycle values (Ct) of target genes were normalized to a reference gene (glyceraldehyde phosphate dehydrogenase; *Gapdh*) and the relative fold changes were determined using the ΔΔCt method. To determine lung viral load, qPCR on IAV polymerase A subunit (*PA*) gene was performed using customed TaqMan probes and normalized to *Gapdh*. Lung viral load is expressed as fold change vs saline controls [23].

### Data and analysis

Data are presented as mean ± SEM except viral load which is presented as median ± interquartile range. All data were analysed using GraphPad Prism 9.0 (Graphpad, San Diego, US). One-way ANOVA with Bonferroni’s post-hoc tests were used. *p* < 0.05 was considered to be statistically significant.

## Results

**Fig. 1:**
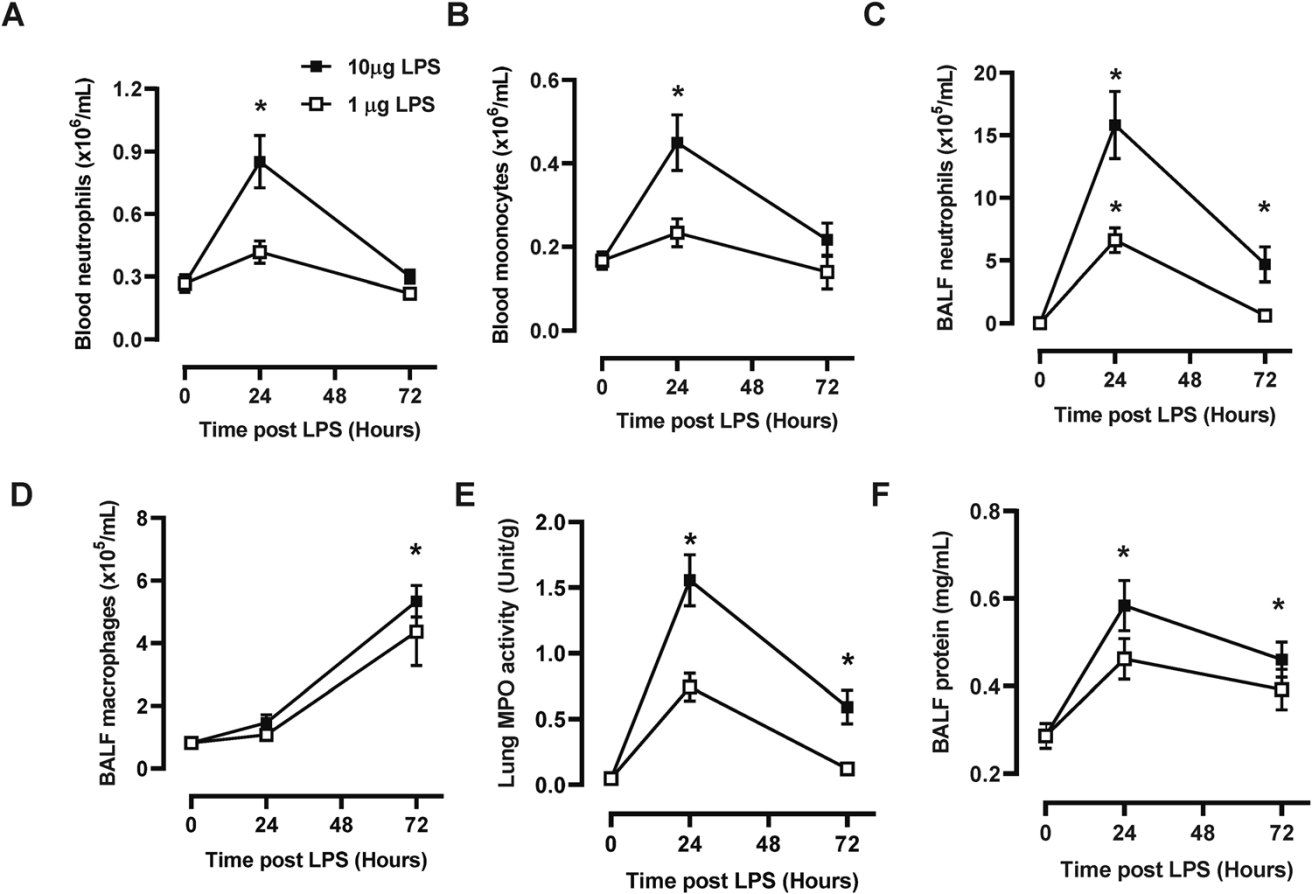
LPS challenge initiates blood myeloid cell expansion and lung leukocyte trafficking. **A** Blood neutrophil and (**B**) monocyte numbers were quantified in hβ_c_Tg mice treated with LPS (1 or 10 μg). **C** BAL neutrophil and (**D**) macrophage numbers were also quantified after LPS challenge (**E**) MPO activity was quantified in lung tissue as a marker for tissue neutrophil infiltration. **F** Total protein levels in BAL-fluid was also measured. *n* = 5–6; data are Mean ± SE; *p < 0.05, one-way ANOVA.

**Fig. 2:**
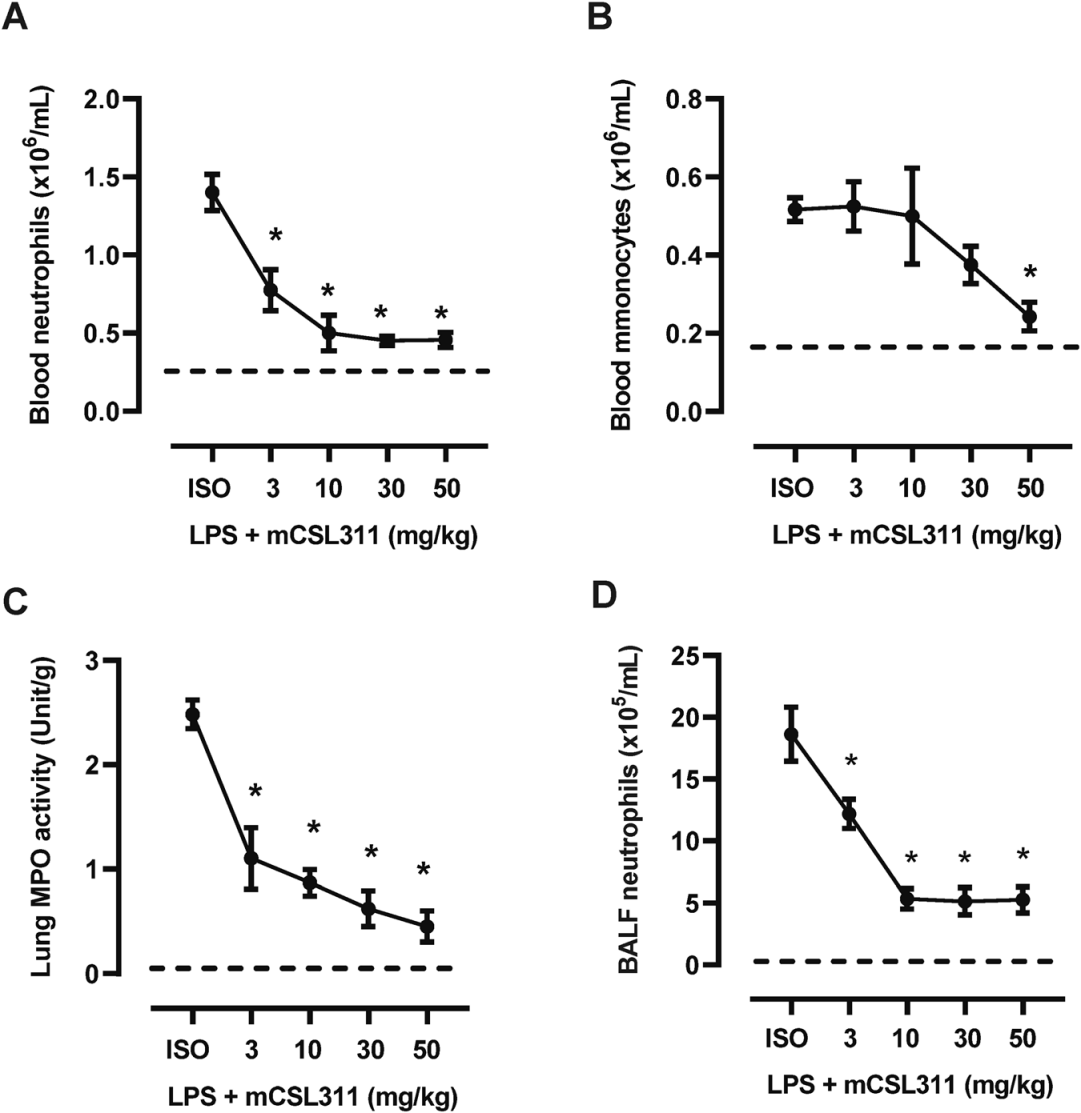
CSL311 dose-dependently reduces myeloid cell numbers in the blood and lungs of hβ_c_Tg mice. **A** CSL311 dose-dependently reduced peak (**A**) blood neutrophil and (**B**) blood monocyte numbers. **C** Peak lung MPO levels declined with maximal decrease observed at the highest 50 mg/kg dose. **D** Peak BAL neutrophil numbers dose-dependently decreased with maximal decrease observed at 10 mg/kg. *n* = 5–6; data are Mean ± SE; *p < 0.05, one-way ANOVA.

LPS challenge resulted in a significant increase in systemic inflammation involving expansion of circulating neutrophil and monocytes, which peaked at 24-hours in hβ_c_ Tg mice treated with the higher dose (10μg) of LPS (Fig. 1A, B). BAL neutrophil numbers tracked with blood neutrophil numbers, where peak numbers were detected in mice treated with high dose LPS at 24-hours, whereas BAL macrophages were only significantly increased at the later 72-hour timepoint (Fig. 1C, D). Lung MPO activity (marker for lung tissue neutrophil infiltration) and BAL fluid protein levels (marker for oedema) peaked at 24-hours following high dose LPS challenge (Fig. 1E, F), which is consistent with blood and BAL neutrophil numbers. We next performed a dose response analysis to identify the optimal dose of CSL311 required to reduce pathological inflammation. CSL311 treatment dose-dependently reduced blood neutrophil numbers with levels declining to control values at the 10 mg/kg CSL311 dose (Fig. 2A). Maximal reduction of peak blood monocyte numbers occurred at the highest 50 mg/kg dose of CSL311 (Fig. 2B). Lung MPO levels were also dose-dependently reduced, with maximal decrease observed at the highest 50 mg/kg dose (Fig. 2C). The suppression of BAL neutrophils closely resembled blood neutrophil numbers, where maximal decrease was observed at 10 mg/kg CSL311 (Fig. 2D).

**Fig. 3:**
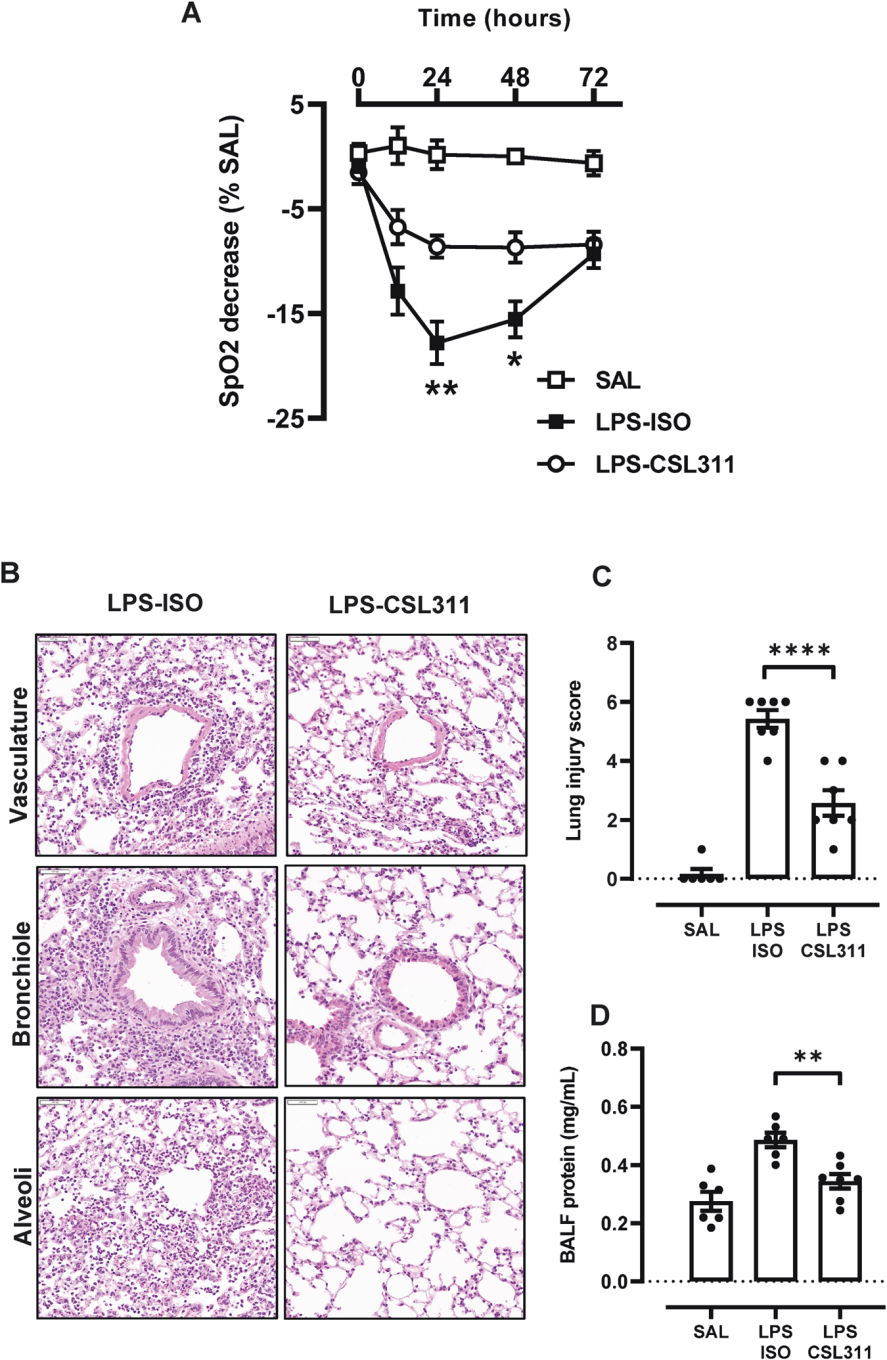
CSL311 improves SpO2 levels by reducing lung inflammation and injury. **A** Blood oxygenation was monitored in hβ_c_Tg mice across multiple timepoints using a MouseSTAT® Jr. Pulse Oximeter, where a single dose of CSL311 significantly improved SpO2 levels that were markedly reduced as a consequence of lung injury caused by LPS challenge. **B** Representative H&E stained lung sections demonstrate that LPS (10 μg at 24-hours) causes inflammation around the vasculature, bronchiole and alveoli. **C** Inflammation was blindly scored, demonstrating that lung injury was significantly reduced with CSL311 treatment. **D** CSL311 treatment also significantly reduced oedema as assessed by quantifying total protein levels in BAL-fluid. *n* = 5–10; data are Mean ± SE; *p < 0.05, **p < 0.005, ****p < 0.0001 ANOVA.

**Fig. 4:**
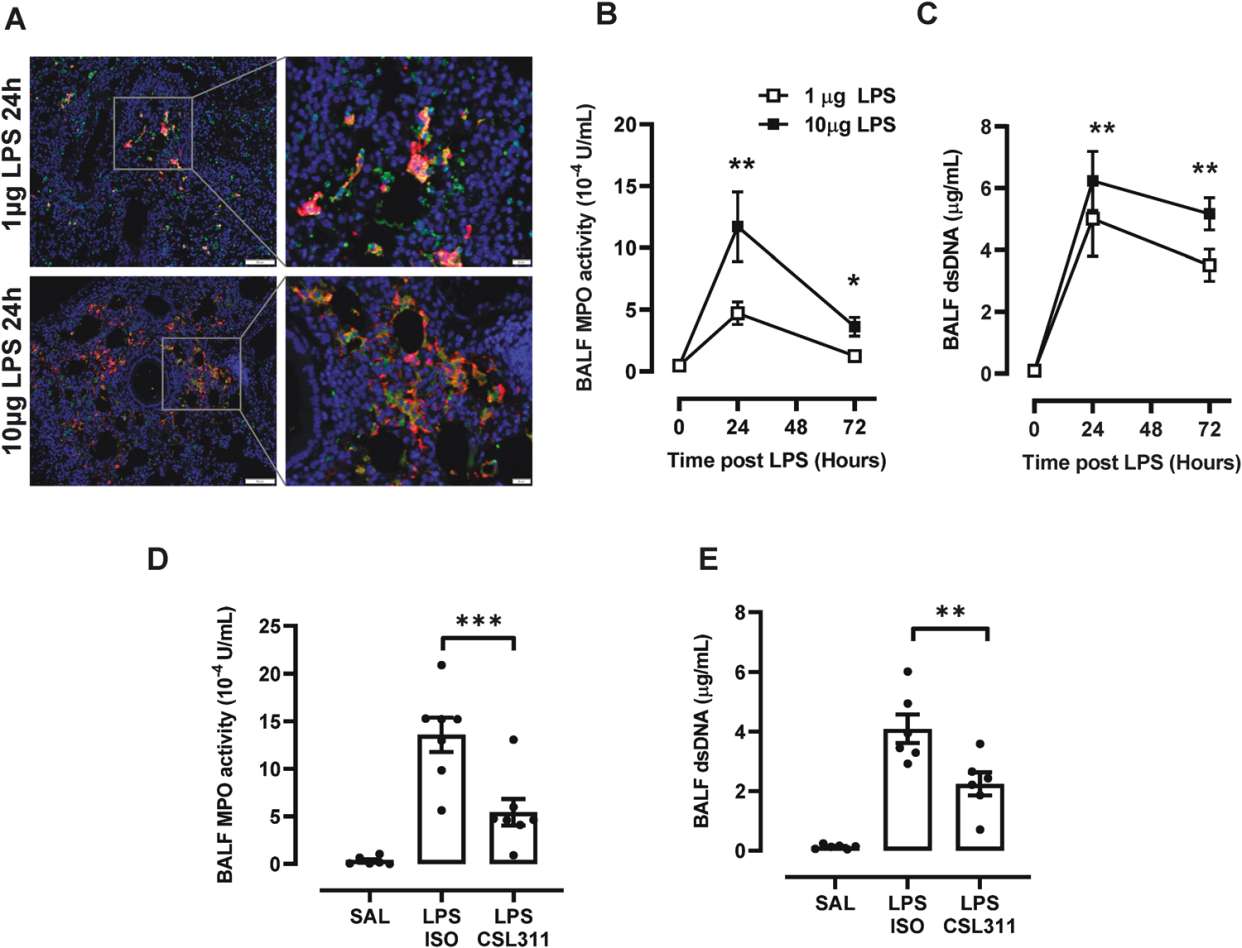
CSL311 significantly reduces netosis in response to LPS challenge in hβ_c_Tg mice. **A** Co-localisation of MPO (green) and histone H3 (red) staining identified lung netosis, which was more prominent with high dose LPS (10 μg). **B** MPO activity and (**C**) dsDNA levels were quantified in the BAL-fluid. **D** MPO activity and (**E**) dsDNA in BALF were significantly reduced with CSL311 treatment. *n* = 6–7; data are Mean ± SE; *p < 0.05, two-way ANOVA.

Oxygen saturation (SpO2) levels were stable in saline treated mice (Fig. 3A). Following LPS challenge, there was a rapid decline in SpO2 levels, peaking at the 24-hour timepoint. Significant improvements in SpO2 were observed in hβ_c_Tg mice treated with a single dose of CSL311 (50 mg/kg). The improvements in respiratory function were driven by the observation that CSL11 treatment markedly reduced lung injury, as assessed by scoring the intensity of vascular, bronchiole and alveolar inflammation in hβ_c_Tg mice (Fig. 3B, C). In addition, CSL311 significantly reduced oedema as determined by quantifying total protein levels in the BAL fluid compartment (Fig. 3D). We next analysed markers of netosis by staining lung tissue sections with antibodies against MPO and citrullinated histone H3. As shown in Fig. 4A, NETs are formed in response to acute LPS challenge (merged orange colour). The exudative NET products were also quantified in the BAL fluid, identifying 24-hours as the peak for detecting MPO and dsDNA levels following LPS challenge (Fig. 4B, C). Peak MPO and dsDNA levels were significantly reduced with CSL311 treatment (Fig. 4D, E).

**Fig. 5:**
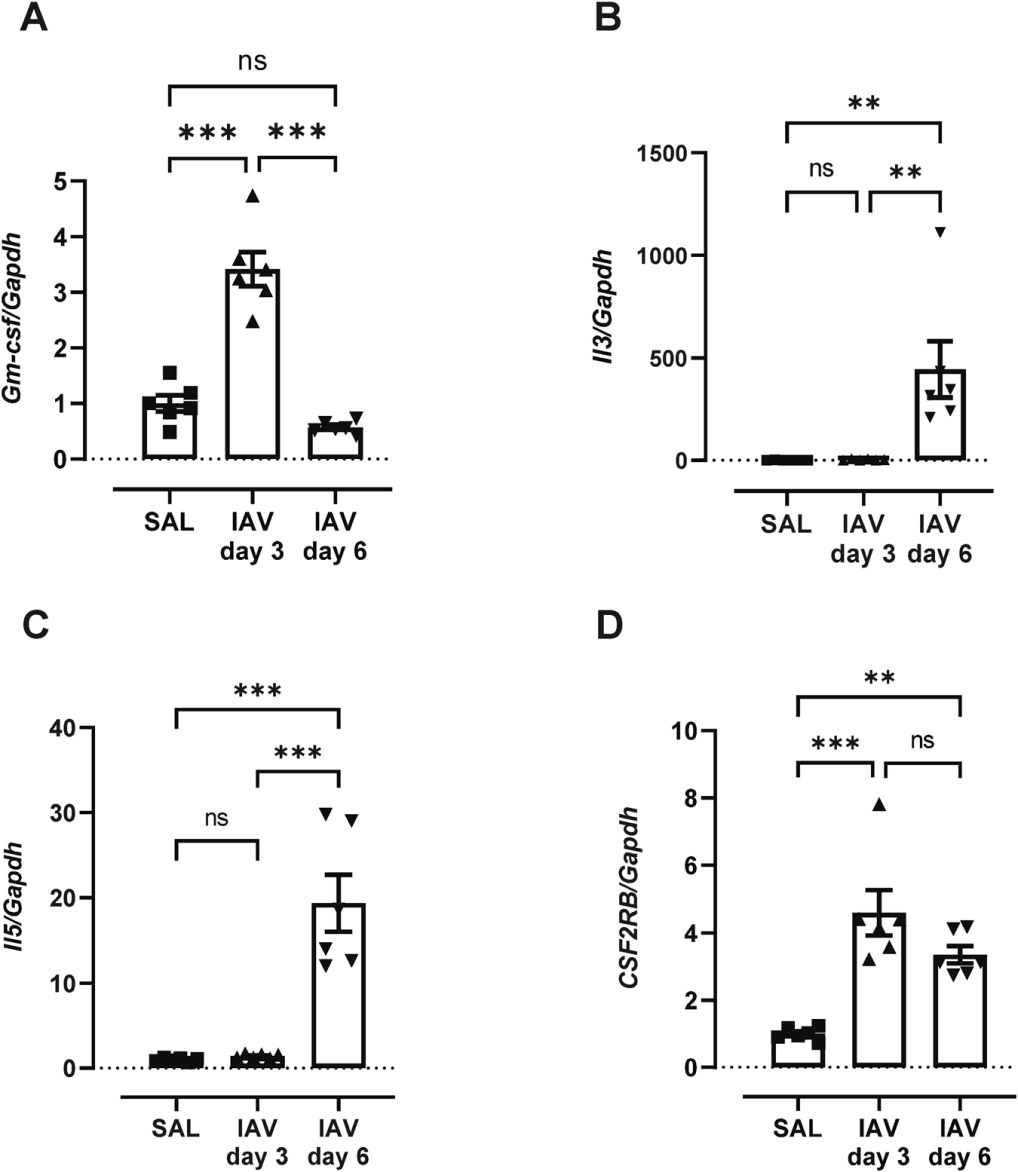
β_c_ cytokines and β_c_ receptor are overexpressed in IAV infected hβ_c_Tg mice. hβcTg mice were infected with IAV (10^4^ PFU, HKx31 strain) and culled at day 3 and day 6 post infection. Gene expression for β_c_ cytokine (**A**) *Gm-csf*, (**B**) *Il3*, (**C**) *Il5* and β_c_ receptor (**D**) *CSF2RB* were analyzed in lung tissue by RTqPCR. *n* = 6; data are Mean ± SE; *p < 0.05, **p < 0.01, ***p < 0.001 one-way ANOVA.

**Fig. 6:**
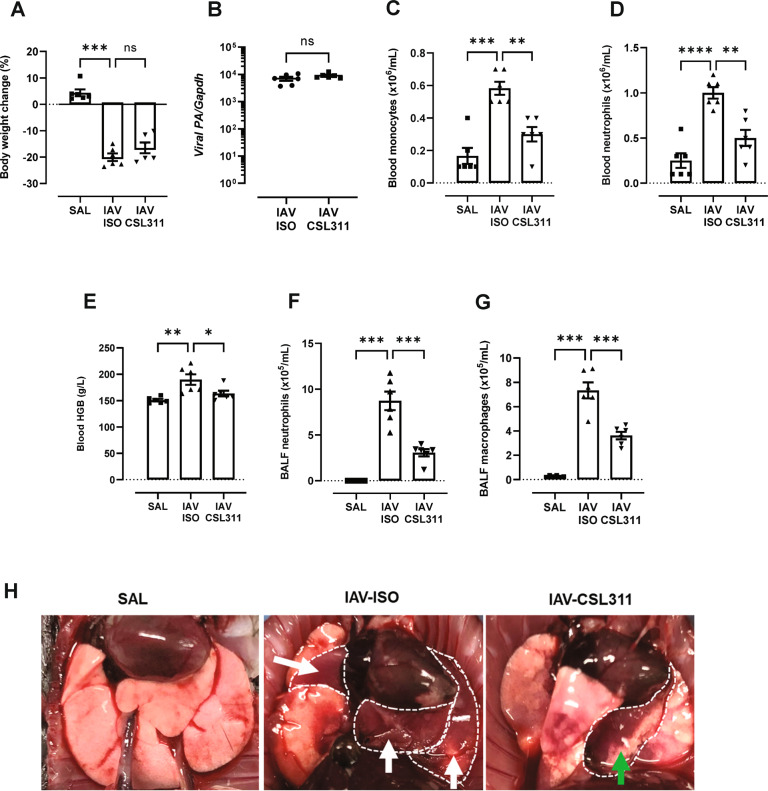
CSL311 reduces lung inflammation and injury without compromising viral clearance in IAV-infected hβ_c_Tg mice. CSL311 or isotype control (ISO) was administered to IAV-infected hβcTg mice at day 4 post infection. At day 7, IAV induced (**A**) body weight loss, which was not significantly improved by CSL311 treatment. (**B**) Lung viral load was measured by RTqPCR on viral *PA* gene and no difference was detected between CSL311 and ISO treated mice infected with IAV. Elevated levels of (**C**) blood monocytes, (**D**) blood neutrophils and (**E**) blood hemoglobin (HGB) induced by IAV infection were significantly reduced by CSL311. **F** BAL neutrophils and (**G**) BAL macrophages were also reduced with CSL311 treatment. **H** Representative photos of lungs identified severe hemorrhage of entire lung lobes (white arrows) in IAV-infected mice, which was less apparent in CSL311 treated mice (green arrow). *n* = 6; data are Mean ±SE except viral loads which are Median ± interquartile ranges; *p < 0.05, **p < 0.01, ***p < 0.001 one-way ANOVA except viral loads which are Mann–Whitney *U* test.

We next inhibited β_c_ receptor signalling in the viral pneumonia setting. Influenza A virus (IAV) lung infection in C57BL/6 mice can largely reproduce the immunopathological features of SARS-COV-2 infection in hACE2 transgenic mice [28]. In hβ_c_Tg mice, *Gm-csf* expression in lung tissue was significantly increased during the early phase of IAV infection (day 3) and declined at day 6 (Fig. 5A). In contrast, *Il3* and *Il5* expression peaked at the later day 6 timepoint (Fig. 5B, C). Expression of the β_c_ receptor transcript (*CSF2RB*) was significantly increased at both day 3 and day 6 post IAV infection, consistent with persistent tissue infiltration of β_c_ receptor-expressing inflammatory cells during acute viral infection (Fig. 5D). A single dose of CSL311 (50 mg/kg) was then delivered to IAV-infected hβcTg mice on day 4 post IAV infection. On day 6, mice infected with IAV displayed a significant reduction in body weight and this was not altered by CSL311 treatment (Fig. 6A). Lung viral loads determined by RTqPCR detected high levels of viral RNA in IAV-infected mice and levels were identical in CSL311 treated mice (Fig. 6B). Blood granulocytosis and monocytosis induced by IAV infection was significantly reduced by β_c_ receptor blockade with CSL311 treatment (Fig. 6C, D). In addition, increased levels of blood haemoglobin (HGB) that is likely to be secondary to IAV-induced hypoxemia [29] was completely prevented by CSL311 treatment (Fig. 6E). Neutrophil (Fig. 6F) and macrophage (Fig. 6G) numbers in the BAL compartment were markedly increased in IAV-infected mice and CSL311 significantly reduced both myeloid cell populations. In addition, IAV infection caused significant haemorrhaging throughout the lung lobes as characterised by the appearance of darkened lung lobes that are plum in colour, as opposed to lungs from saline treated mice that were pink in colour. CSL311 treatment was associated with a reduction in haemorrhagic regions throughout the lung lobes (Fig. 6H).

**Fig. 7:**
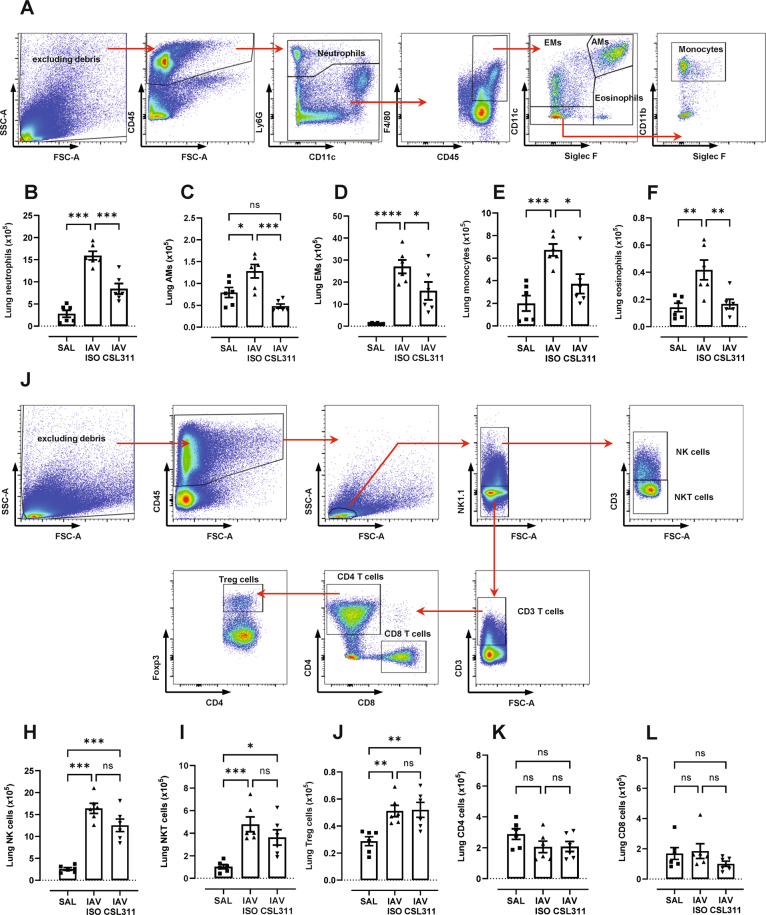
CSL311 blocks lung myeloid cells and preserves NK cell and T cells in IAV-infected hβ_c_Tg mice. Flow cytometry analysis utilizing a (**A**) gating strategy for myeloid cells revealed that lung (**B**) neutrophils, (**C**) alveolar macrophages (AMs), (**D**) exudative macrophages (EMs), (**E**) monocytes and (**F**) eosinophils were highly increased in hβcTg mice at day 6 post IAV infection, and significantly reduced with CSL311 treatment. Flow cytometry analysis utilizing a (**G**) gating strategy for delineating lymphoid cells demonstrated that lung (**H**) NK cells, (**I**) NKT cells and (**J**) regulatory T cells (Tregs) were increased following IAV infection and were not affected by CSL311 treatment. **K** Lung CD4 cells and (**L**) CD8 cell numbers in the lungs did not differ across all groups; *n* = 6; data are Mean ± SE; *p < 0.05, **p < 0.01, ***p < 0.001 one-way ANOVA.

To further characterise immunological changes in the lungs following CSL311 treatment of IAV infected hβ_c_Tg mice, myeloid (Fig. 7A) and lymphoid cells (Fig. 7J) were analysed by flow cytometry. Lung neutrophils (Fig. 7B) and macrophages (Fig. 7C-E) were significantly reduced in in IAV-infected mice following CSL311 treatment, consistent with changes observed in the blood and BAL. The reduction in macrophage numbers was driven by a decrease in alveolar macrophage numbers (AMs, Fig. 7C), monocyte derived exudative macrophages (EMs, Fig. 7D) and blood monocytes (Fig. 7E) recruited into the lungs. Lung eosinophil numbers, previously reported to increase in IAV-infected mice [30], were also increased in IAV-infected hβ_c_Tg mice and βc receptor antagonism with CSL311 markedly reduced this response (Fig. 7F). In contrast to myeloid cells, lymphocyte subsets that confer vital protection against viral infections do not directly respond to βc cytokines. A marked expansion of natural killer (NK) cells (Fig. 7H) and NKT cells (Fig. 7I) was observed at day 6 post IAV infection, which was not significantly altered by CSL311 treatment. Immunosuppressive Regulatory T (Treg) cells were increased by IAV infection in a manner that was not affected by CSL311 treatment (Fig. 7J). Lung CD4 and CD8^+^ T cells were not increased at day 6 post IAV infection and CSL311 treatment did not further alter their respective numbers. (Fig. 7K, L).

**Fig. 8:**
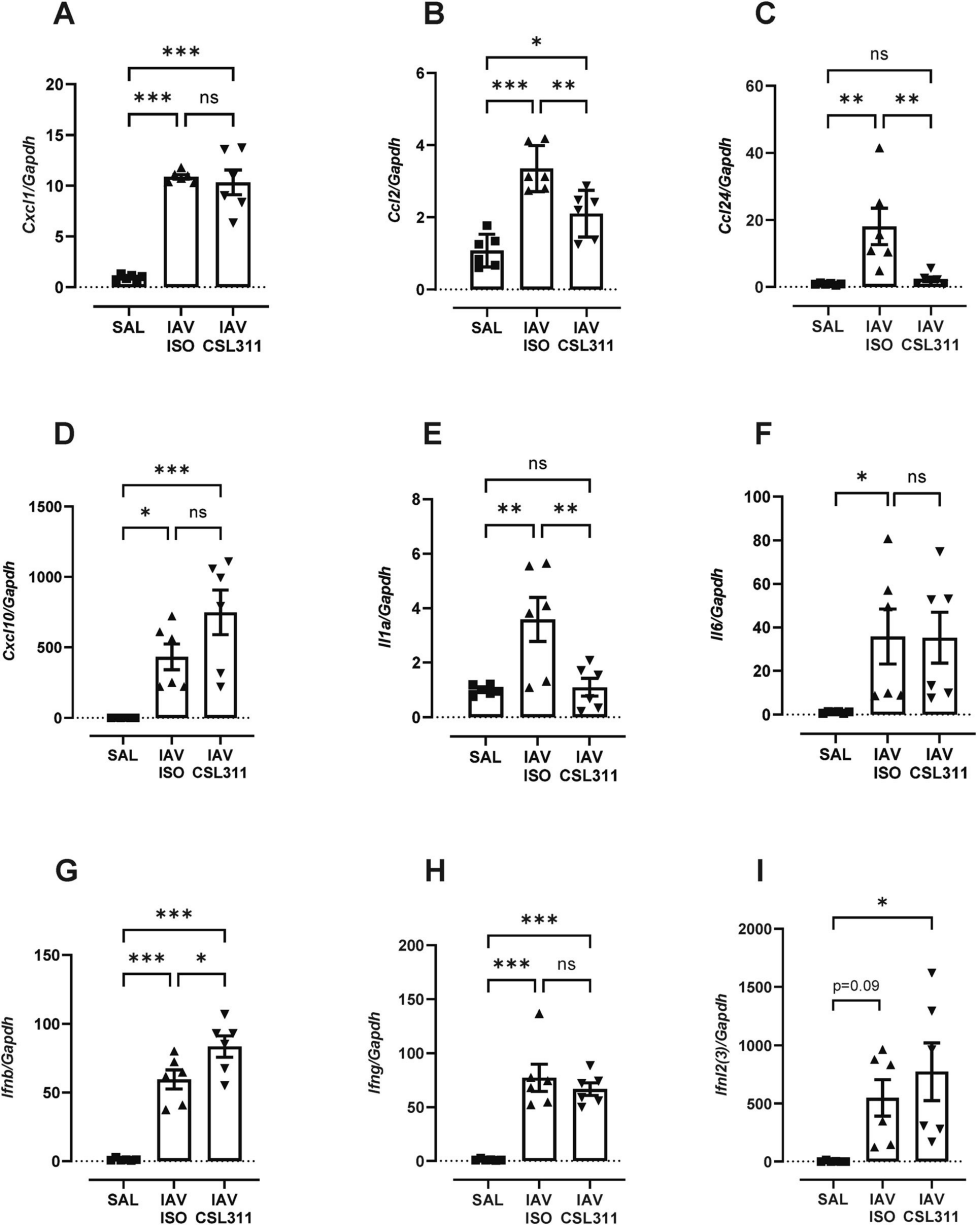
CSL311 attenuates lung cytokine storm and preserves interferon expressions in IAV-infected hβ_c_Tg mice. Expression of the neutrophil chemokine (**A**) *Cxcl1*, monocyte/macrophage chemokine (**B**) *Ccl2*, eosinophil chemokine (**C**) *Ccl24*, T cell/NK cell chemokine (**D**) *Cxcl10* were detected by RTqPCR in the lungs of IAV-infected mice, where CSL311 significantly lowered *Ccl2* and *Ccl24* levels. Expression of the pro-inflammatory genes (**E**) *Il1a* and (**F**) *Il6* were also increased in IAV-infected mice and CSL311 treatment significantly reduced *Il1a* but not *Il6* expression. **G** Type I interferon (*Ifnb*), (**H**) type II interferon (*Ifng*) and (**I**) type III interferon (*Ifnl2/3*) were also markedly induced in the lungs of IAV-infected mice in a manner that was not significantly altered by CSL311 treatment; *n* = 6; *p < 0.05, data are Mean ± SE; **p < 0.01, ***p < 0.001 one-way ANOVA.

At a molecular level, lung expression of the neutrophil chemokine *Cxcl1*, the monocyte/macrophage chemokine *Ccl2* and the eosinophil chemokine *Cc124* were significantly increased in IAV-infected mice (Fig. 8A–C). CSL311 treatment did not reduce *Cxcl1* expression but did significantly reduce *Ccl2* and *Ccl24* expression. These reductions are consistent with lung macrophages being a major cellular source of CCL2 and CCL24 [30] during IAV infection, which are reduced by CSL311 treatment. *Cxcl10* has several roles including chemotaxis of NK and NKT cells, and its levels were increased in IAV-infected mice in a manner that was not altered by CSL311 treatment (Fig. 8D). *Cxcl1* and *Cxcl10* can be produced by various cell types including fibroblasts and endothelial cells [31, 32], which are not targets for βc antagonism. The inflammatory cytokine *Il1α* was increased in the lungs of IAV-infected mice and βc receptor blockade completely normalised *Il1α* levels (Fig. 8E), which is consistent with IL1α being mainly produced by exudative macrophages [33]. IL-1R signalling can regulate expression of neutrophil adhesion molecules and the reduction in IL-1α may contribute to reduced neutrophilic inflammation in the lungs. *Il6* expression was also increased in IAV-infected mice and its levels were not reduced in CSL311 treated mice (Fig. 8F). Interferons constitute the first line of anti-viral defence and all three types of interferons were upregulated in IAV-infected lungs (Fig. 8G–I). Type II interferon (*Ifng*) levels were not altered by CSL311 treatment, consistent with cellular sources (NK/NKT cells and cytotoxic T cells) not being altered by CSL311 treatment. IAV-induced expression of type I (*Ifnb)* and type III interferons (*Ifnl2/3*) were also preserved in CSL311-treated mice (Fig. 8G, I), which suggests that their cellular sources (plasmacytoid dendritic cells (pDCs) and lung epithelia) are functionally competent in the background of β_c_ receptor antagonism.

## Discussion

Biologics that target GM-CSF for COVID-19 pneumonia or ARDS are actively being investigated in clinical trials, including TJ003234 (NCT04341116), Lenzilumab (NCT04351152), Gimsilumab (NCT04351243) and Mavrilimumab (NCT04447469) [34]. Our strategy of targeting the human β_c_ receptor with CSL311 has the distinct advantage of not only blocking GM-CSF signalling, but also suppressing IL-5 signalling. IL-5 is biologically active during respiratory viral infections, where NKT cells and alveolar macrophages produce IL-33, which subsequently enhance IL-5 production from innate lymphoid cells (ILC2) [35]. The production of IL-5 in response to influenza infection stimulates the accumulation of eosinophils in the lungs, which can cause eosinophilic pneumonia acutely [15, 36] but may also contribute to the clearance of virus [37]. We observed increased *Il5* lung expression in our viral pneumonia model that was associated with a significant increase in lung eosinophil numbers at day 6 post IAV infection. Importantly, we demonstrate that β_c_ receptor antagonism markedly reduced eosinophil numbers in the lungs to control levels without increasing viral loads.

IL-5 levels and eosinophil numbers have been reported to be systemically elevated in severe COVID-19 patients [13]. Conversely, a reduction in total circulating eosinophils was detected in severe COVID-19 patients during the later phases of infection [38]. The cause of peripheral eosinopenia is not known but may reflect the active migration of eosinophils into the target tissue [39] or may be attributed to eosinophilic apoptosis triggered by corticosteroid treatment. Eosinophils have been detected in the alveolar interstitium of deceased SARS-CoV-2 patients [14] and case reports have described pulmonary eosinophilic vasculitis in a severe COVID-19 patient [40]. It is plausible that toxic proteins and mediators released from activated eosinophils in the lungs can contribute to the pathogenesis of viral pneumonia when in excess. IL-5 signalling can also stimulate neutrophil-dependent responses during respiratory viral infections via the IL-5Rα expressed on migrated neutrophils [41]. Hence, there is a mounting case for targeting IL-5 signalling, which appears to be a safe strategy based on the use of type-2 therapeutics in asthmatics with SARS-Cov-2 infection [42].

We also utilised an acute LPS lung injury model involving excessive neutrophil airway recruitment to demonstrate that CSL311 is very effective in preventing lung injury, oedema, and respiratory distress. Additionally, we demonstrate that β_c_ receptor antagonism reduced the formation of NETs, which can contribute to mucosal and vascular injury and cell death [7–9]. The generation of NETs can be controlled by a form of regulated cell death known as necroptosis mediated by RIPK3 (receptor-interacting protein kinase 3) and MLKL (mixed lineage kinase domain-like) [43]. Furthermore, necroptosis is a molecular driver of pathological inflammation and airway remodelling in lung diseases [44]. Hence, CSL311 may represent a novel strategy to block necroptosis and NETs, however further work is needed to define the interaction between βc receptor signalling, necroptosis, and NET generation.

Additionally, we tested CSL311 in a viral pneumonia model to investigate whether blockade of β_c_ receptor signalling may compromise essential host immunity to pathogens during serious respiratory infections. We demonstrate that the therapeutic delivery of CSL311 markedly reduced systemic and lung inflammation without significantly altering lung viral loads, lung NK/NKT cells or expression of type I, II and III interferons. Whilst we did not specifically investigate myeloid-derived suppressor cells (MDSCs), they can expand and differentiate in response to GM-CSF [45]. MDSCs are capable of suppressing IAV-specific immune responses resulting in higher viral IAV titres, and importantly, NKT cells induced during IAV infection actively suppress MDSCs [46]. Our findings suggest that CSL311 may also inhibit expansion of MDSC populations without altering NKT cell expansion to support anti-viral immunity, although further work is needed to confirm this.

We observed that lung *Gm-csf* expression was increased during the early phase of infection (day 3) where it can coordinate an immunological response to the pathogen, and we initiated CSL311 treatment at day 4. Pre-clinical mouse studies demonstrate that the prophylactic administration of recombinant GM-CSF can be protective against IAV infection [47] and in patients, increased levels of GM-CSF during the early phase of ARDS is associated with improved prognosis [48]. Sargramostim is a recombinant human GM-CSF therapeutic recently trialled in COVID-19 patients (NCT04326920) as a strategy to improve blood oxygenation. However, it is plausible that treatment with recombinant GM-CSF in patients with established viral pneumonia and hyperinflammation may have limited efficacy during the later phases of infection. Our findings demonstrate that the therapeutic delivery of CSL311 safely reduced inflammation in mice with established viral pneumonia.

The role of IL-3 also requires further investigation in the context of severe COVID-19 pneumonia and ARDS. In a hyperoxia-induced acute lung injury model, the genetic depletion of IL-3 conferred protection by reducing the degree of inflammation, injury and oedema [49]. IL-3 has also been identified as an orchestrator of emergency myelopoiesis during sepsis, where IL-3 deficiency reduced pathological inflammation and injury in mice. Of significance, hβ_c_Tg mice do not respond to IL-3 as the mouse Il-3Rα subunit may not form a functional signalling complex with the human βc receptor [17]. Our data demonstrates that hβ_c_Tg mice mount a robust immunological response to both LPS and IAV, to suggest that IL-3 signalling has a limited role in the LPS or IAV setting due to compensatory mechanisms including expression of GM-CSF and other cytokines that can stimulate myelopoiesis. In addition, since the type I and type III interferons are largely derived from pDCs during viral infections, their production in hβcTg mice suggests that IL-3 may have a limited role in pDC expansion during influenza infection.

In summary, we have utilised a unique transgenic mouse model expressing the human β_c_ receptor subunit to show that blocking β_c_ receptor signalling with CSL311 markedly decreased circulating myeloid expansion and trafficking into the lung upon LPS challenge and IAV infection. Consequently, the extent of myeloid cell accumulation and lung injury was significantly reduced in CSL311-treated hβ_c_Tg mice. Based on these findings, CSL311 should be considered as an enhanced therapeutic strategy to combat myeloid cell-mediated immunopathology caused by ALI/ARDS or severe viral pneumonia. The ability of CSL311 to block GM‐CSF, IL-5 and IL‐3 signalling has the potential to simultaneously reduce pathological inflammatory and type-2 immunity in various disease settings where immunity is maladapted. The complexity of immunity to SARS-CoV-2 infection represents a disease setting where single target therapeutics may have limited utility on a population basis. We suggest that a therapeutic strategy targeting multiple inflammatory cytokines simultaneously (illustrated here with CSL311) has the potential to reduce the inflammatory storm more effectively than single target therapeutics.

### Reporting summary

Further information on research design is available in the Nature Research Reporting Summary linked to this article.

## Supplementary information

reporting summary

## Data Availability

The datasets used and/or analyzed during the current study are available from the corresponding author on reasonable request.
